# Affective Modulation after High-Intensity Exercise Is Associated with Prolonged Amygdalar-Insular Functional Connectivity Increase

**DOI:** 10.1155/2020/7905387

**Published:** 2020-03-25

**Authors:** Angelika Schmitt, Neeraj Upadhyay, Jason Anthony Martin, Sandra Rojas Vega, Heiko Klaus Strüder, Henning Boecker

**Affiliations:** ^1^Functional Neuroimaging Group, Clinic for Diagnostic and Interventional Radiology, University Hospital Bonn, Venusberg-Campus 1, Building 7, 53127 Bonn, Germany; ^2^German Center for Neurodegenerative Diseases, Venusberg-Campus 1, Building 99, 53127 Bonn, Germany; ^3^Institute of Movement and Neurosciences, German Sport University, Am Sportpark Müngersdorf 6, 50933 Cologne, Germany

## Abstract

Acute moderate exercise has been shown to induce prolonged changes in functional connectivity (FC) within affect and reward networks. The influence of different exercise intensities on FC has not yet been explored. Twenty-five male athletes underwent 30 min of “low”- (35% < lactate threshold (LT)) and “high”- (20% > LT) intensity exercise bouts on a treadmill. Resting-state fMRI was acquired at 3 Tesla before and after exercise, together with the Positive and Negative Affect Scale (PANAS). Data of 22 subjects (3 dropouts) were analyzed using the FSL feat pipeline and a seed-to-network-based analysis with the bilateral amygdala as the seed region for determining associated FC changes in the “emotional brain.” Data were analyzed using a repeated measures ANOVA. Comparisons between pre- and post-exercise were analyzed using a one-sample *t*-test, and a paired *t*-test was used for the comparison between “low” and “high” exercise conditions (nonparametric randomization approach, results reported at *p* < 0.05). Both exercise interventions induced significant increases in the PANAS positive affect scale. There was a significant interaction effect of amygdalar FC to the right anterior insula, and this amygdalar-insular FC correlated significantly with the PANAS positive affect scale (*r* = 0.47, *p* = 0.048) in the “high”-intensity exercise condition. Our findings suggest that mood changes after exercise are associated with prolonged alterations in amygdalar-insular FC and occur in an exercise intensity-dependent manner.

## 1. Introduction

Physical exercise diminishes symptoms of depression and anxiety [1, 2], and regular exercise training has been suggested as an additive nonpharmacological treatment strategy for affective disorders [3]. Imaging methods, in particular functional magnetic resonance imaging (fMRI), have now started to identify the associated changes in brain functional states induced by short- and long-term exercise interventions in reward [4–6] and mood [7, 8] processing areas. In some of these initial studies, prolonged changes in neuronal activity were identified after exercise challenges using resting-state fMRI (rs-fMRI) in the affect and reward network (ARN): Weng et al. reported a significant increase in resting-state functional connectivity (rs-FC) in the ARN after a single bout of moderate exercise on a bicycle ergometer (compared to a passive control condition) in older and younger healthy volunteers [8]. However, findings were not directly related to measures of individual exercise-induced affective states. To date, imaging data specifically probing exercise-induced affective modulation have only been sampled in long-term interventions: Tozzi et al. [7] investigated 38 healthy sedentary volunteers in their midforties and randomized them into an aerobic exercise group or a control group. Subjects underwent a longitudinal study design with a training duration of 16 weeks. They recorded mood changes and rs-fMRI over time to identify the underlying functional network changes linked to the mood-related benefits of exercise. The authors reported increased rs-FC between the parahippocampal gyrus and areas involved in mood regulation (the left supramarginal gyrus, the left superior temporal gyrus, the right superior temporal pole, and the left precentral area) in the exercise group, but not in the control group. These results suggest that pertinent rs-FC changes mediate the benefits in a mood induced by regular physical activity. Moreover, we have previously investigated the acute effects of different exercise intensities on FC using an ICA-based whole brain approach [9]. An increase in rs-FC in the frontoparietal network after low-intensity exercise was described, whereas high-intensity exercise resulted in increased ARN rs-FC and decreased sensorimotor rs-FC.

What is so far missing in the literature are experimental studies focusing on the neuronal underpinnings of prolonged modulatory effects on a mood following acute exercise bouts. Moreover, the issue of exercise intensity-dependent functional connectivity (FC) changes and their relation to affective states remain largely enigmatic, thus constituting the direct subject matter of this experimental study. Here, we aimed to investigate the effects of two defined exercise intensities (“high” and “low,” adapted to individual fitness levels) on rs-FC between the amygdala and regions of the “emotional brain” [10] in order to probe whether and how exercise intensity affects amygdalar rs-FC, in a seed-to-network approach. Based on initial work reporting exercise-induced alterations in the ARN [7–9] and the integral role of the amygdala within this network [11], we predicted exercise-induced changes in amygdalar-ARN rs-FC which are (a) exercise intensity dependent and (b) linked with a positive mood.

## 2. Methods

The reported experimental procedure and the MRI data acquisition parameters are identical to the published study by Schmitt et al. [9].

### 2.1. Participants

We recruited twenty-five right-handed healthy recreational male athletes via social media and flyers at the university and sport clubs. None of the subjects were professional athletes. While some subjects were training for a triathlon, most of them were pure recreational athletes performing their training on an individual basis. All subjects were frequently runners and exercised in this discipline at least 3 times/week for 45 min for the past two years. Subjects were without any prior history of head injury, neurological or psychiatric illness, and orthopedic or general health problems that may have prohibited physical activity. Other exclusion criteria were MRI contraindications (e.g., metal and/or electronic implants and claustrophobia). Three of 25 subjects had to be excluded from our final analyses due to injuries (caused by private activities, *N* = 1) and not finishing the study (*N* = 2), resulting in a final sample size of *N* = 22 subjects. Descriptive characteristics including age, education, verbal intelligence level, and handedness of participants were acquired from a set of questionnaires (sociodemographics, Edinburgh Handedness Inventory [12], and German vocabulary test [13]) (Table 1). Furthermore, psychiatric questionnaires were administered, including the Mini International Neuropsychiatric Interview (MINI, German version 5.0.0) [14], the State-Trait Anxiety Inventory (STAI) [15], and the Beck Depression Inventory (BDI) [16]. None of the 22 participants showed results indicating psychiatric diseases (MINI, BDI, and STAI). The BDI and STAI state inventories were acquired on each visit before the examinations started. BDI scores did not differ significantly between examination days. STAI state scores on the other hand showed a significant difference between examination days (“low”: 28.9 ± 5.3, “high”: 32.1 ± 7.8; *t*(21) = −2.71; *p* = 0.013). To rule out potential effects of factors that show significant differences between examination days, these were inserted as covariates of no interest in statistical analyses (see below).

Participants were well informed about the study, and written informed consent was obtained after detailed explanation of all tests, potential discomforts, risks, and procedures employed in the investigation. The study was approved by the local ethics committee of the University Hospital Bonn (Ethikkommission an der Medizinischen Fakultät der Rheinischen Friedrich-Wilhelms-Universität Bonn: Nr. 340/13), according to national legislation and the Declaration of Helsinki.

### 2.2. Experimental Procedure

All participants completed three within-subject experimental sessions in randomized order (high, low, and self-selected). Each session was separated by at least two days, but a maximum of 12 weeks. Prior to this, all participants underwent a preexperimental session at the German Sport University Cologne including medical history, stethoscopy, a 12-channel resting electrocardiogram (ECG; MAC 1200 ST, GE Medical Systems Information Technologies GmbH, Freiburg, Germany), and an incremental exercise test on a treadmill (Woodway PPS Med, Woodway GmbH, Germany) to assess individual health and fitness. The initial speed of the incremental exercise test was set at 5 km/h, with a fixed gradient of 1%. Exercise intensity/running speed was increased to 1 km/h every 3 min. Participants were asked to perform until volitional exhaustion. Capillary blood samples (20 *μ*L) were collected from the earlobe to measure blood lactate concentration within the last 30 s of each stage. Additionally, the heart rate (HR; POLAR, Kempele, Finland) was monitored and a rating of perceived exertion (RPE) [17] was recorded at the end of each stage. Ratings on the RPE scale varied from "6-no exertion at all/extremely light" to "20-maximal exertion". Maximal effort was considered to be achieved with the attainment of at least two of the following criteria: high levels of blood lactate concentration (8–10 mmol/L), a perceived rate of exertion of ≥18, and/or a HR of ±10 beats per minute (bpm) of age-predicted maximum (220-age) [18]. Blood lactate samples from the incremental exercise test were analyzed using the Biosen C-Line lactate analyzer (EKF Diagnostics GmbH, Barleben, Germany). The lactate threshold (LT) was determined via visual inspection as the first sustained increase in blood lactate above baseline values [19].

All three experimental sessions consisted of a 30 min exercise bout at either “low,” “high,” or “self-selected” exercise intensity on a treadmill in randomized order to exclude order effects. Each exercise bout started with a 5 min warmup at 5 km/h, followed by the main 30 min exercise intervention, and later ended with a 5 min cool down period at 5 km/h. Resting blood pressure was recorded before and after each exercise intervention. HR, physical exertion, and blood lactate concentration were acquired before, during, and after each exercise intervention. Blood lactate and physical exertion were recorded during the intervention every 5 min, while HR was recorded every minute.

The “low”-intensity exercise was performed at 35% under LT to ensure a very easy exercise condition that challenges primarily the aerobic energy supply. The exercise intensity for the “high” condition was set at 20% above LT primarily demanding the anaerobic energy supply and accumulating lactate. PANAS ratings and fMRI scans were acquired on all three examination days directly pre- and post-exercise. The rs-fMRI sequence was acquired approx. 30 min post-exercise bouts (after the acquisition of a CASL sequence and before additional fMRI sequences and structural images). To account for possible prolonged effects of exercise on resting HR, HR was monitored during MRI scans using a pulse oximetry transducer placed on the left index finger (BIOPAC Systems Inc., Goleta, CA, USA). Data was recorded using the software of BIOPAC (AcqKnowledge 4.1).

To avoid any influence of exercise-induced fatigue or stimulating effects of diet, participants were instructed to refrain from intense exercise and alcohol intake 24 hours prior to each testing session as well as from caffeine and meals two hours before the experiment. As this manuscript focuses exclusively on the comparison of standardized and metabolically defined exercise intensities, data from the self-selected intervention will be reported elsewhere.

### 2.3. MRI Acquisition

The MRI examination took place at the Department of Radiology (University Hospital Bonn) using a 3 T clinical MRI System (Ingenia 5.1.7, Philips Healthcare, Best, the Netherlands), equipped with an 8-channel head coil. A rs-fMRI sequence was acquired using a T2^∗^-weighted gradient echo, echo-planar imaging (EPI) protocol sensitive to the blood oxygenation level-dependent (BOLD) contrast with 250 volumes: TR = 2595 ms, TE = 35 ms, FOV = 230 × 230 × 147 mm, image matrix = 64 × 64, flip angle = 90°, 3.59 × 3.59 × 3.59 mm^3^ voxels, and interleaved acquisition (1, 3, 5, 7…41; 2, 4, 6, 8…40; first odd and then even) of 41 contiguous axial slices of 3.59 mm thickness. For all rs-fMRI scans, participants were instructed to lay still for 11 min with their eyes closed and to stay awake without focusing on any particular thought. All images were acquired parallel to the anterior-posterior commissure plane with no interslice gap. An anatomical 3D T1-weighted sequence was also acquired within each run using the following parameters: slice orientation: sagittal, acquisition matrix: 256 × 256, acquired voxel size: 1 × 1 × 1 mm^3^, sequence type: 3D FFE, TR: 7.6 ms, TE: 3.9 ms, flip angle: 15°, and total scan duration: 4:39 min.

### 2.4. Data Preprocessing

To compare pre- to post-exercise and between-condition changes in a common space, structural images were first averaged from all time points to create a mean image for each participant [20] using SPM12 (Statistical Parametric Mapping; Wellcome Department of Imaging Neuroscience, London, UK) implemented in MATLAB (The Mathworks Inc., Sherborn, MA, USA). Functional and structural images were evaluated using the FSL toolbox version 5.0.9 (the FMRIB Software Library, https://www.fmrib.ox.ac.uk/fsl). Preprocessing of the functional images included motion correction, brain extraction, and spatial smoothing with 6 mm FWHM (full width at half maximum). Affine linear and nonlinear registration was performed to register the functional images from each subject to their mean structural images.

A robust ICA-based strategy for Automatic Removal of Motion Artifacts (ICA-AROMA) was used to further remove physiological noise and high-frequency components preserving autocorrelations. Later, we regressed out time series signals from possible sources of interference, i.e., white matter (WM) and cerebrospinal fluid (CSF). Afterwards, we band-pass filtered the data to remove frequencies other than 0.009 to 0.08 Hz. The denoised filtered functional data from every participant's pre- and post-exercise images including “high” and “low” exercise conditions were transformed to the MNI152 template (2 mm^3^ resolution) space.

### 2.5. Creating a Seed and Network Mask

After the preprocessing, a seed-to-network analysis was performed. Therefore, we created our seed and network masks using the Wake Forest University PickAtlas toolbox [21]. We chose the bilateral amygdala as our seed region and two separate masks for the left and right hemispheres encompassing all brain areas (beyond the amygdala) of the “emotional brain”: the anterior cingulum, middle cingulum, posterior cingulum, inferior orbitofrontal cortex, medial orbitofrontal cortex, middle orbitofrontal cortex, superior orbitofrontal cortex, dorsolateral prefrontal cortex, hypothalamus, insula, nucleus accumbens, and pallidum [9].

In order to evaluate the changes pre- to post-exercise and “high” versus “low” exercise intensity, seed-to-network-based connectivity maps were created for all individual participants in each intervention condition: this was done by calculating correlations between the seed regions' mean time course and the network mask of the left and right emotional brain [22]. Fisher's *r* to *z* transformation was then performed to create the respective FC maps, for both the pre- and the post-sessions of the rs-fMRI in each condition.

### 2.6. Statistical Analysis

All statistical analyses of behavioral data were performed using SPSS 24 (SPSS Inc., Chicago, Illinois).

#### 2.6.1. Physiological Data

A paired *t*-test was used to analyze the exercise-related parameters and to compare the exercise conditions (“low” vs. “high”) with each other. The analyzed parameters were as follows: average speed over 30 min of exercise intervention, maximum HR, average HR over 30 min of exercise intervention, maximum blood lactate, and maximum RPE.

Using a repeated measures 2 × 2 ANOVA (with the factor condition (“low”/“high”) and time (pre/post-exercise)), HR during the MRI session was analyzed. Therefore, mean HR was calculated for every single session. Due to artifacts in the HR recordings, only 18 subjects have been included in the ANOVA. Additionally, post hoc paired *t*-tests with the Bonferroni correction were performed. Significant results were reported at *p* values of less than 0.05.

#### 2.6.2. PANAS

Testing the PANAS data for normal distribution using the Shapiro-Wilk test revealed that the data of the negative affect scale is not normally distributed. However, simulation studies have shown that repeated measures ANOVA is relatively robust against violations of normal distribution assumptions, if it is the only assumption that has been violated [23]. Therefore, the PANAS was analyzed by entering the values into a 2 (intervention :  ^“^low^”^/^“^high^”^) × 2 (time : pre/post-exercise) × 2 (scale : positive affect scale/negative affect scale) repeated measures ANOVA. The positive affect scale and the negative affect scale were then entered separately into a 2 (intervention :  ^“^low^”^/^“^high^”^) × 2 (time : pre/post-exercise) repeated measures ANOVA. Afterwards, paired *t*-tests with the Bonferroni correction were performed. *p* values of less than 0.05 were regarded as statistically significant.

#### 2.6.3. Functional Connectivity

We applied a general linear model to evaluate the modulation of FC between the bilateral amygdala seed region and the left and right “emotional brain” network after exercise in each condition (pre- vs. post-exercise) and between conditions (“high” vs. “low”). First, we created difference maps (post minus pre) for both exercise conditions within each subject. Then, a one-sample *t*-test was applied on these individual difference maps to measure within-condition effects. To compare conditions with each other (“low” vs. “high”), a paired *t*-test design with individual difference maps as an input was applied. Additionally, we performed the same analyses again but including the following covariates of no interest: HR during the scanning session, maximum blood lactate in the exercise intervention and STAI state acquired at the beginning of each testing day. Due to missing HR data, the one-sample *t*-test with covariates included *N* = 20 subjects for “low” and “high.” As the two subjects excluded for the “low” condition were not the same that had to be excluded in the “high” condition, the analysis for comparing exercise conditions (“low” vs. “high”) included only *N* = 18 subjects. Modulations were assessed by randomizing each component per subject using nonparametric 5,000 random permutations, incorporating a threshold-free cluster enhancement (TFCE) technique [24]. Significant clusters at a statistical threshold of *p* < 0.05 were identified using the Harvard-Oxford atlas implemented in FSL [25]. Results reported are corrected for multiple comparisons (Bonferroni correction).

#### 2.6.4. Correlation Analysis

In order to investigate the association between the FC changes in the “emotional brain” and the behavioral data (PANAS), a two-tailed correlation analysis was performed for each exercise intervention separately. Therefore, the *β*-values of the significant clusters (of our analyses with the three covariates of no interest) were extracted at the level of each subject. The delta *β*-values and the delta PANAS values (postexercise minus preexercise) were used for the correlation analysis.

## 3. Results

### 3.1. Acute Exercise Manipulation

Mean HR increased during the “low” exercise intervention from resting HR of 62.0 ± 6.3 bpm to 119.2 ± 15.8 bpm and from resting HR of 65.5 ± 12.1 bpm to 176.8 ± 6.9 bpm in the “high” exercise intervention. The paired *t*-test revealed that the mean HR during the exercise conditions differed significantly between the two exercise conditions (*t*(21) = −15.3, *p* < 0.001). Moreover, the maximum HR also differed significantly between the two exercise intensities (“low”: 128.2 ± 17.2 bpm, “high”: 186.5 ± 6.5; *t*(21) = −16.5; *p* < 0.001). We also found significant differences for perceived exertion (“low”: 9.2 ± 2.0; “high”: 18.5 ± 1.3; *t*(21) = −22.0, *p* < 0.001), speed (“low”: 7.1 ± 0.7 km/h, “high”: 12.8 ± 1.3 km/h; *t*(21) = −22.0; *p* < 0.001), and blood lactate concentration (“low”: 2.8 ± 1.9 mmol/L, “high”: 10.2 ± 3.2 mmol/L; *t*(21) = −10.6; *p* < 0.001). Both physiological and subjective measures indicate that our experimental design was based on two significantly different exercise intensities.

### 3.2. Physiological Monitoring during the fMRI Scan

For the 2 × 2 repeated measures ANOVA, *N* = 4 subjects needed to be excluded from the analysis due to artifacts (sample size: *N* = 18). Results revealed a significant main effect of condition (*F*(1, 17) = 41.92, *p* < 0.001), a main effect of time (*F*(1, 17) = 115.93, *p* < 0.001), and a significant time × condition interaction (*F*(1, 17) = 82.24, *p* < 0.001). In the post hoc testing, *N* = 2 of 22 subjects needed to be excluded for the “low” condition and also *N* = 2 for the “high” condition, resulting in a sample size of *N* = 20 subjects. Comparisons showed a significant increase in HR after the “high” exercise bout compared to preexercise (*t*(19) = −14.99, *p* < 0.001; pre-“high”: 58.9 ± 9.0 bpm, post-“high”: 78.0 ± 9.6 bpm). No significant change in HR was found from pre- to post-“low” exercise (*t*(19) = −0.88, *p* = 0.390; pre-“low”: 57.7 ± 8.1 bpm, post-“low”: 58.6 ± 8.7 bpm). Comparing the exercise conditions with each other (delta of post- minus pre-“low” vs. post- minus pre-“high”) also revealed a significant effect (*t*(17) = 9.07, *p* < 0.001); i.e., the increase from pre- to post-“high” (19.1 ± 5.7 bpm) was significantly greater than the increase from pre- to post-“low” (1.0 ± 5.5 bpm). Baseline measurements (pre-exercise) did not differ from each other (*t*(19) = −0.65, *p* = 0.524).

### 3.3. PANAS

Analyzing the PANAS values revealed a significant main effect of time (*F*(1, 21) = 31.38, *p* < 0.001), a significant main effect of scale (*F*(1, 21) = 228.69, *p* < 0.001), and a significant time × scale interaction (*F*(1, 21) = 17.00, *p* < 0.001) in the 2 × 2 × 2 ANOVA.

**Figure 1:**
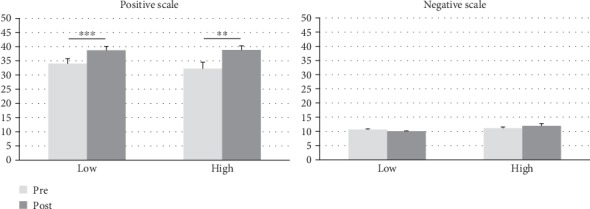
PANAS values measured pre/post-“low” and pre/post-“high” exercise. *N* = 22. Error bars indicate the standard error of the mean. ^∗∗∗^*p* < 0.001, ^∗∗^*p* < 0.01.

The 2 × 2 ANOVA for the positive affect scale showed a significant main effect of time (*F*(1, 21) = 27.09, *p* < 0.001). Post hoc comparisons of the positive affect scale revealed a significant increase for both exercise interventions: pre- (34.0 ± 8.5) to post- (38.7 ± 6.6) “low”-intensity exercise (*t*(21) = −5.47, *p* < 0.001) and pre- (32.3 ± 10.6) to post- (38.8 ± 6.9) “high”-intensity exercise (*t*(21) = −3.94, *p* = 0.001) (Figure 1).

The analysis of the negative affect scale only revealed a significant main effect of condition (*F*(1, 21) = 6.29, *p* = 0.020). Comparing the two exercise conditions post hoc showed no significant differences between the conditions “low” and “high”.

### 3.4. Amygdalar FC within the “Emotional Brain”

#### 3.4.1. Bilateral Amygdala to the Right Hemisphere “Emotional Brain” Network

**Figure 2:**
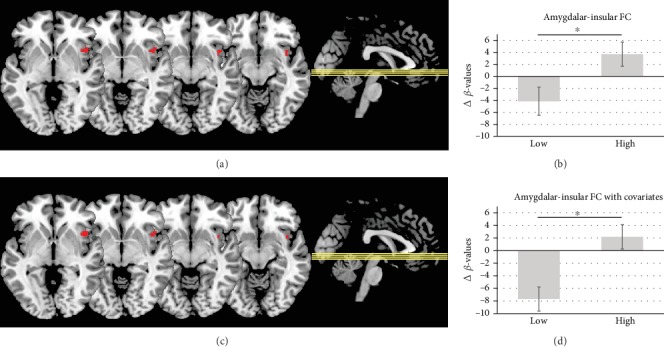
Representation of the observed significant change in amygdalar-insular FC between conditions (“low” and “high”). (a) In analysis without covariates (*N* = 22), there is peak activation in the right insula in [46 6 -2] (cluster size *k* = 56). (b) Change in FC as delta *β*-values (post minus pre) in analysis without covariates. (c) In analysis with STAI state, blood lactate, and HR as covariates of no interest (*N* = 18), there is peak activation in the right insula in [44 16 -2] (cluster size *k* = 54). (d) Change in FC as delta *β*-values (post minus pre) in analysis with STAI state, blood lactate, and HR as covariates. ^∗^*p* < 0.05, TFCE. Clusters are overlaid onto a single-subject MNI template for visualization; error bars indicate the standard error of the mean.

Our FC analysis between the bilateral amygdala seed and the right hemisphere “emotional brain” network revealed a significant interaction effect in the right anterior insula (AI) (*p* = 0.031): by extracting the *β*-values of the significant cluster of the AI, we detected that the interaction effect was driven by an increase in FC after the “high”-intensity exercise intervention and a decrease in FC after the “low”-intensity exercise intervention (Figures 2(a) and 2(b)). However, directly comparing pre- versus post-“low”-intensity exercise and pre- versus post-“high”-intensity exercise did not reveal significant differences.

#### 3.4.2. Bilateral Amygdala to the Right Hemisphere “Emotional Brain” Network with Covariates

The FC analysis with three covariates of no interest (HR, blood lactate, and STAI state) further supported our results without covariates, despite the smaller sample size (*N* = 18). Comparing “low” and “high” exercise intensity also revealed a significant change in FC in the right insula (*p* = 0.021). This interaction was also driven by a decrease in FC after the “low”-intensity exercise intervention and an increase in FC after the “high”-intensity exercise intervention (Figures 2(c) and 2(d)). Directly comparing pre- versus post-“low”-intensity exercise and pre- versus post-“high”-intensity exercise did not reveal significant differences.

#### 3.4.3. Bilateral Amygdala to the Left Hemisphere “Emotional Brain” Network

The FC analysis between the bilateral amygdala seed and the left hemisphere “emotional brain” network revealed no significant differences in FC with or without covariates.

### 3.5. Correlation between Amygdalar-Insular Connectivity and the PANAS Positive Affect Scale

**Figure 3:**
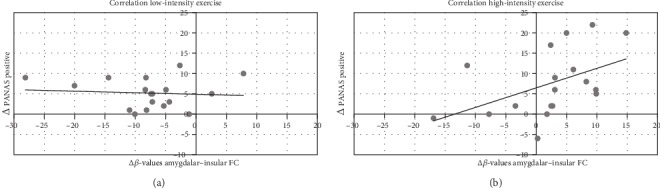
Correlation between the delta of post minus pre FC of the amygdala to the right AI and delta of post minus pre PANAS positive scores: (a) in the “low” condition; (b) in the “high” condition. *N* = 18.

Analysis revealed a significant positive correlation between the change in the PANAS positive affect scale and the increase in amygdalar-insular FC after the “high”-intensity exercise intervention (*r* = 0.471, *p* = 0.048; Figure 3(b)). In the “low”-intensity exercise condition, we found no significant correlation (*r* = −0.084, *p* = 0.740; Figure 3(a)).

## 4. Discussion

This is the first study to investigate the differential effects of individually titrated “low”- and “high”-intensity aerobic exercise bouts specifically on the FC in emotional brain regions, i.e., between the amygdala and areas belonging to the “emotional brain” using rs-fMRI in healthy young recreational athletes. While generally supporting initial evidence of rs-FC increases in affective brain regions that persist after exercise, the results of this study also provide novel evidence of exercise intensity-dependent modulatory effects on rs-FC, namely, by demonstrating a significant interaction effect (time × condition) in amygdalar-insular FC: this was driven by an increase in FC between the amygdala and the right AI after the “high”-intensity exercise intervention, while a decrease was found after the “low”-intensity exercise intervention. This increase in amygdalar-insular FC after “high”-intensity exercise was correlated with the change in the PANAS positive affect scale, suggesting that exercise-induced amygdalar-insular FC increases are associated with positive mood changes.

Assessments of mood changes revealed a significant increase in the positive affect scale after both exercise interventions (“low” and “high”), which is in line with previous literature [26, 27]. No significant differences were identified in the negative affect scale. Pre-exercise, the values of the negative affect variable were already near the bottom of the range (floor effect).

Our investigations were *a priori* centered on the “emotional brain” as described by Dalgleish, who emphasizes a special role of the amygdala [9, 28] but also other interconnected areas involved in emotion processing (prefrontal cortex, cingulate cortex, hypothalamus, and insula). The prominent role of the amygdala within the “emotional brain” was traditionally linked to fear processing [29]; currently, however, a much broader functional role of the amygdala for the processing of different salient stimuli has been proposed [30], such as for the processing of social cues, in particular faces [31, 32], independent of cue valence [33]. Anatomically, the amygdala is densely and reciprocally interconnected with the prefrontal cortex, posterior cingulate cortex, precuneus, parietal and occipital lobes, insula, thalamus, and striatum [34, 35]. Evidence from multiple methodologies across species supports a central role for the “amygdala hub” within a large-scale neural network that makes up the emotional/social brain [36].

Findings provide independent supporting evidence to a study by Weng et al. [8] that identified a significant within-network rs-FC increase in the left amygdala and the right AI after a moderate exercise bout, however, without reporting behavioral measures of mood. Our data indicate that amygdalar-insular FC changes depend on the intensity of the exercise bout, suggesting specific exercise intensities to regulate emotion processing in a differential manner.

The AI plays an important role for processing emotions, including social ones like empathy and compassion [37], but also interpersonal phenomena like fairness and cooperation [38]. Neuroimaging studies suggest that the AI is involved in the evaluative, experimental, and expressive aspects of specific individual emotions, such as happiness, sadness, fear, and disgust [39]. These multiple aspects of emotional processing may be attributed to the diverse anatomical connections of the AI [40, 41].

Structural and/or functional changes in amygdalar-insular FC were identified in different psychiatric conditions, including anxiety disorders (posttraumatic stress disorder, phobia, and panic) [42–45], depression [46–48], schizophrenia [49–51], and autism [52–54]. Exercise has already been shown to have a positive effect on depression and anxiety symptoms [1, 2], and fMRI studies have shown abnormal activity in the insula and the amygdala in patients with depression [55, 56] and anxiety disorders [57]. While the majority of studies have shown decreased amygdalar-insular rs-FC in depressed patients [58–62], two studies found increased FC [63, 64]. Reduced FC of the amygdala with regions involved in emotional processing, as found in the majority of studies, may lead to abnormal affect regulation in depression and may be considered a functional state marker reflecting impaired bottom-up signaling for top-down cortical modulation of limbic regions [61]. The literature on amygdalar-insular FC in anxiety disorders is less clear: some studies reported greater amygdalar-insular FC in patients with generalized anxiety disorder [65], while posttraumatic stress disorder patients show decreased amygdalar-insular FC [66].

Our findings suggest a distinct neural mechanism underlying positive affective modulations after strenuous exercise [26, 27]. The correlation between the change in the PANAS positive affect scale and the change in amygdalar-insular FC in the “high” exercise condition validates the role of amygdalar-insular FC for affective modulation and the impact of exercise intensity thereon. Physical exercise appears to strengthen amygdalar-insular rs-FC, leading to an improved mood and reduced fear, a concept that will have to be validated experimentally in dedicated patient populations in the future. Potentially, these effects are mediated by endogenous opioid release, given that positron emission tomography ligand activation studies were able to report local exercise-induced opioid release in the AI [67–69] after exercise.

There are some limitations that must be considered in the current study. To exclude potential hormone cycle-linked humoral factors, only male subjects were included. Future studies should investigate gender-balanced cohorts. Additionally, no control intervention was implemented, so it cannot be concluded with certainty that effects shown in the pre- vs. post-comparisons are also driven by factors other than exercise. We consider this to be unlikely, as the change of amygdalar-insular rs-FC induced by exercise is generally in line with previous initial findings in the literature (as mentioned above). Although we already acquired affective measures (PANAS), future studies might include further affective questionnaires that capture a wider emotional range. Moreover, it was not always possible to measure both conditions for one subject at the same time during the day, due to restricted availability of the MRI scanner and the subjects. Future studies should try to account for this issue. Finally, we have to acknowledge that our study does not allow any firm conclusions on why “low” exercise and “high” exercise were associated with opposite effects, rather than scaled effects on FC in particular networks. This issue will have to be further investigated by incorporating a control condition (no exercise) and additional measurements of excitatory and inhibitory neurotransmission, for instance, with PET radioligand studies or MR spectroscopy, which could be implemented in hybrid PET-MR systems.

## Figures and Tables

**Table 1:** Participants' characteristics.

Variable	*M* ± SD
Age (years)	27.1 ± 4.0
Height (cm)	182 ± 6
Weight (kg)	78.3 ± 6.8
BMI (kg/m^2^)	23.7 ± 1.4
HR_max_ (bpm)	194.2 ± 7.2
Peak running speed (km/h)	16.1 ± 1.3
Running speed at LT (km/h)	10.7 ± 1.1
Education (years)	17.9 ± 2.7
GVT	106.4 ± 9.8
EHI	83.4 ± 15.1^∗^
BDI	1.1 ± 1.9
STAI trait	30.8 ± 6.2

BMI = body mass index; BDI = Beck Depression Inventory (score ≤ 9: no depression); EHI = Edinburgh Handedness Inventory; HR = heart rate; GVT = German vocabulary test; LT = lactate threshold; STAI = State-Trait Anxiety Inventory (range: 20 = not being afraid to 80 = maximum intensity of anxiety). ^∗^*N* = 21, due to one subject that was ambidextrous (laterality quotient: 21.74); *N* = 22.

## Data Availability

The data generated and analyzed during this study are available on request from the corresponding author. Due to the containing information that could compromise the privacy of research participants, the data are not publicly available.
